# Functional and structural connectivity success predictors of real-time fMRI neurofeedback targeting DLPFC: Contributions from central executive, salience, and default mode networks

**DOI:** 10.1162/netn_a_00338

**Published:** 2024-04-01

**Authors:** Daniela Jardim Pereira, João Pereira, Alexandre Sayal, Sofia Morais, António Macedo, Bruno Direito, Miguel Castelo-Branco

**Affiliations:** Neurorradiology Functional Area, Imaging Department, Coimbra Hospital and University Center, Coimbra, Portugal; Coimbra Institute for Biomedical Imaging and Translational Research (CIBIT), University of Coimbra, Coimbra, Portugal; Faculty of Medicine, University of Coimbra, Coimbra, Portugal; Institute of Nuclear Sciences Applied to Health (ICNAS), University of Coimbra, Coimbra, Portugal; Siemens Healthineers Portugal, Lisboa, Portugal; Psychiatry Department, Coimbra Hospital and University Center, Coimbra, Portugal; Instituto do Ambiente, Tecnologia e Vida (IATV), Coimbra, Portugal

**Keywords:** Neurofeedback, Connectivity, Success, Prediction, rt-fMRI, DTI

## Abstract

Real-time functional magnetic resonance imaging (rt-fMRI) neurofeedback (NF), a training method for the self-regulation of brain activity, has shown promising results as a neurorehabilitation tool, depending on the ability of the patient to succeed in neuromodulation. This study explores connectivity-based structural and functional success predictors in an NF *n*-back working memory paradigm targeting the dorsolateral prefrontal cortex (DLPFC). We established as the NF success metric the linear trend on the ability to modulate the target region during NF runs and performed a linear regression model considering structural and functional connectivity (intrinsic and seed-based) metrics. We found a positive correlation between NF success and the default mode network (DMN) intrinsic functional connectivity and a negative correlation with the DLPFC-precuneus connectivity during the 2-back condition, indicating that success is associated with larger uncoupling between DMN and the executive network. Regarding structural connectivity, the salience network emerges as the main contributor to success. Both functional and structural classification models showed good performance with 77% and 86% accuracy, respectively. Dynamic switching between DMN, salience network and central executive network seems to be the key for neurofeedback success, independently indicated by functional connectivity on the localizer run and structural connectivity data.

## Author Summary

Neurofeedback using real-time fMRI (rt-fMRI NF) is a potential neurorehabilitation tool for several neurodevelopmental, psychiatric and neurological disorders. However, not all subjects are capable to learn how to self-regulate their brain, that is, to be successful in rt-fMRI NF. Our goal, in this study, is to find connectivity metrics that predict this success in a working memory paradigm targeting dorsolateral prefrontal cortex (DLPFC), so that we can avoid futile implementation of the technique in the future by previously selecting responders. We found that success is probably dependent on the dynamic switching between DMN, salience network, and central executive network, influenced by both functional and structural connectivity.

## INTRODUCTION

Neurofeedback (NF) using real-time fMRI (rt-fMRI) is a technique that allows an individual to learn how to control brain activity, by providing immediate information about one’s blood oxygen level–dependent (BOLD) signal in a specific brain region or network. However, it is estimated that about 38% of the subjects are not able to modulate brain activity based on neurofeedback (Haugg et al., 2020), often referred to nonresponders in the NF literature. This variability in the response to NF depends on both methodological and individual factors, as reviewed by Stoeckel et al. (2014), Sitaram et al. (2017), Thibault & Raz (2017), and Fede et al. (2020). Previous research proved that intrinsic factors like brain structure (Zhao et al., 2021) and brain function prior to NF training (Haugg et al., 2020; Nakano et al., 2021; Scheinost et al., 2014; Skouras & Scharnowski, 2019) may potentially be used as subject-specific predictors of neurofeedback success.

In the NF literature, we still lack a gold standard measure for success (Haugg et al., 2020, 2021; Paret et al., 2019; Stoeckel et al., 2014; Thibault et al., 2018). The assumed time scale of training effects, pattern of change (linear vs. nonlinear, monotonic vs. non-monotonic), and participants’ individual differences are some of the factors we should take into account when defining a success metric (Stoeckel et al., 2014). As critically reviewed by Paret et al. (2019), possible NF training success indices are simple comparisons between rest and task such as fixed threshold, success rate, personal effect size or mean performance. However, these measures do not translate/represent participants’ improvement over time. To overcome this issue, possible success metrics are calculated between the last and first session (but any change between these time points will get lost) or estimated with a learning slope, which will account for temporal dynamics, even though it often assumes a linear improvement that might not be true in NF performance (Sitaram et al., 2017). The slope of the neurofeedback learning curve was used in both meta-analyses from Haugg et al. (2020, 2021), since it allows comparisons across different studies in a way that all participants are pooled together. This is a critical aspect when searching for predictors extendable to different paradigms and populations.

The first study that aimed at predicting NF success based on brain function (Scheinost et al., 2014) performed a whole-brain connectivity analysis of a pretraining resting-state fMRI and found a significant correlation between behavioral improvement and higher connectivity in orbital frontal cortex/BA 10, which was coincident with the NF target region of interest (ROI). Also, based on pre-NF resting-state scans from a large database, Skouras and Scharnowski (2019) proved that the ability to learn self-regulation of default mode network (DMN) was partially determined by eigenvector centrality of posterior cingulate cortex (PCC)/precuneus (PC) (Skouras & Scharnowski, 2019). Similarly, PCC-based connectivity showed to be a predictor of NF aptitude (the ability to change brain activity through NF training), independently of the NF target region (Nakano et al., 2021). A recent meta-analysis (Haugg et al., 2020) failed to find a pre-NF training brain-based predictor that was common to all types of studies included (different target ROI, NF method, and participants condition). However, they demonstrated a slight predictive power of target ROI activity levels on the functional localizer run, but not in the no-feedback runs and only when considering as success metric the difference between last and first NF runs (Haugg et al., 2020).

Studies investigating structural MRI data as predictors of NF success are even more scarce. The voxel-based morphometry (VBM) study from Zhao et al. (2021) demonstrated an association between larger volumes of putamen and NF learning success. More recently, Caria and Grecucci (2023), in a supervised machine learning study, showed that several gray matter and white matter regions predict real-time fMRI based anterior insula regulation. However, they did not find any regional volumetric measure predictive of NF learning success. In the broader field of brain-computer interfaces (BCI), another study explored structural predictors of the potential skill in using EEG-BCI interfaces based on VBM and diffusion tensor imaging (DTI) (Halder et al., 2013). They achieved a 93.75% accuracy in classifying a participant as belonging to the high- or low-aptitude group based on DTI data, but not with VBM. To our knowledge, structural connectivity (DTI) was not previously assessed as a possible predictor for neurofeedback success.

These studies investigating potential predictors based on pretraining functional connectivity used resting-state fMRI (Nakano et al., 2021; Scheinost et al., 2014; Skouras & Scharnowski, 2019), which was added to the NF protocol and thus extended the duration of the MR acquisition. Furthermore, both the relevance of the activation patterns in the localizer run and the potential predictive value of its connectivity measures were emphasized in the meta-analyses from Haugg and colleagues (Haugg et al., 2020, 2021), but they lacked sufficient data to analyze these aspects.

In our study, we investigate possible functional connectivity predictors detectable on the task-based localizer run, which is already included in most neurofeedback protocols, without the need to extend the MR acquisition. Additionally, we explore for the first time the predictive value of structural connectivity for rt-fMRI neurofeedback performance. Furthermore, we performed a machine learning approach to test the predictive value for both structural and functional connectivity metrics. Finally, we attempt to establish conceptual links between both connectivity metrics, which also represents a novel contribution in the NF literature.

## METHODS

### Participants

Seventeen healthy volunteers were included in this study (10 male, mean age 27.8 ± 4.2 years). All had normal or corrected-to-normal vision, were right-handed, and had no history of neurological or psychiatric diseases. All gave informed written consent before participating, in accordance with the declaration of Helsinki, and the study complied with the safety guidelines for magnetic resonance imaging (MRI) research on humans. The work was approved by the Ethics Committee of the Faculty of Medicine of the University of Coimbra.

### Data Acquisition

MRI data were acquired on a 3T Siemens Magnetom TrioTim scanner with a 12-channel head coil. Functional imaging is based on an echo planar imaging (EPI) sequence with 32 slices, in-plane resolution: 3 × 3 mm, FOV: 192 × 210 mm, matrix 64 × 70, slice thickness: 2.5 mm, FA: 75°, TR = 2,000 ms, and TE = 30 ms. Diffusion-weighted images were acquired using a spin EPI sequence in contiguous axial planes (TR = 8,020 ms, TE = 83 ms, matrix size = 128 × 128, FOV = 256 × 256 cm^2^, slice thickness = 2 mm without interslice gaps, 64 slices for whole-brain coverage) with diffusion sensitization gradients applied in 64 noncollinear directions, with a b value of 700 s/mm^2^. For reference anatomical runs, we used a high-resolution magnetization-prepared rapid acquisition gradient echo (MPRAGE) sequence (176 slices; echo time (TE): 3.42 ms; repetition time (TR): 2,530 ms; voxel size: 1 × 1 × 1 mm; flip angle (FA): 7°; field of view (FOV): 256 × 256 mm).

The MRI session included reference anatomical images, DTI and a neurofeedback real-time functional protocol, which consisted in a localizer run followed by five imagery runs targeting the dorsolateral prefrontal cortex (DLPFC), the first (train) and last (transfer) without providing feedback information to the participant. The scanning session lasted approximately 1 hour and 30 minutes.

### Experimental Neurofeedback Protocol

The localizer task consisted of a *n*-back working memory paradigm with a ‘1-back’ (stimulus matched the one before) and a ‘2-back’ (stimulus matched one presented two stimuli before) conditions distributed randomly in 10 blocks (5 blocks per condition), alternating with baseline blocks, being the total length of the run 10 minutes and 30 seconds. The visual stimulus was created and presented on Presentation 20.1 (Neurobehavioral Systems, Inc.).

The imagery runs included two conditions—‘Imagery’ and ‘Baseline’—presented alternatively six times per run with an additional ‘Baseline’ block at the beginning of each run, each condition block lasting 30 seconds. Visual feedback of mean target ROI activation (updated every TR) was provided in the form of a thermometer divided into 10 levels, each representing a given percent BOLD signal change. Participants were instructed to empty the thermometer during ‘Baseline’ conditions and increase the thermometer bars during the ‘Imagery’ condition, through a cognitive strategy of backward reciting the self-generated numeric sequences subvocally. They were also instructed to adjust the content, length, and difficulty of the sequences they generated and the speed of recitation according to the feedback.

### Online ROI Definition

We functionally targeted the DLPFC using the real-time fMRI software package Turbo-BrainVoyager 3.2 (TBV; Brain Innovation, Maastricht, The Netherlands). Real-time preprocessing included 3D head motion correction (6 degrees of freedom) and an intrasession alignment, using the first volume of the localizer as a reference for all functional data, to correct for head movements across runs. Online statistical analysis of incoming volumes was incremental, using a recursive least squares general linear model (GLM) based on a design matrix automatically created from the imported stimulation protocol. Activation clusters were mainly estimated according to the contrast ‘2-back’ > ‘baseline’, complemented by the contrast ‘2-back’ > ‘1-back’ to identify the effort-related clusters associated with the task and delimitate a more precise ROI. The activation cluster also had to respect anatomical references: anterior to premotor cortex and superior to the planes, including lateral ventricles. All targets were selected on the left hemisphere since participants performed a verbal working memory task during imagination runs (Emch et al., 2019).

### Offline fMRI Data Analysis for Individual Target ROI

Offline fMRI data analysis was performed using BrainVoyager QX 2.8 (Brain Innovation, Maastricht, The Netherlands) following standard preprocessing steps, including slice scan time correction, 3D motion correction, temporal high-pass filtering (GLM Fourier method, two cycles), spatial smoothing using a 3D gaussian kernel (full width half maximum = 6 mm), and normalization to Talairach (TAL) coordinate space. First-level analysis was performed using a GLM for each run. The design matrix included a predictor for each experimental condition, and confound predictors for the six motion parameters (three translational and three rotational) and motion spikes (volumes with root mean square displacement greater than 0.25).

To evaluate DLPFC modulation we computed the *t* value for the contrast of interest (‘Imagery’ > ‘baseline’) for all imagery runs in the target region and used it as the measure of the participant’s ability to modulate the target region.

Based on previous literature (Haugg et al., 2020, 2021; Thibault et al., 2018), we defined neurofeedback success as the linear trend on the *t*-values for the contrast of interest of the three NF runs. To this end, we estimated a linear regression model (optimized using least squares fit) using MATLAB (MathWorks). A positive linear trend in this metric would represent an improvement, accounting for learning dynamics during the NF runs. To assess if modulation of the target region, during the localizer, was related with the target’s regulation during NF runs, we used a linear regression model.

### Functional Connectivity Data Analysis

Since the goal of this study was to investigate potential pretraining neuroimaging predictors, our functional connectivity analysis was focused on the localizer run data, considering the conditions primarily used for target definition: ‘baseline’ and ‘2-back’ (higher signal amplitude due to cognitive load compared to the intermediate 1-back condition). Data were preprocessed and analyzed using the CONN toolbox version 21a (Nieto-Castanon, 2021). Preprocessing pipeline comprised functional realignment, slice-timing correction, outlier identification, direct normalization into MNI space, segmentation into gray matter, white matter, and CSF and spatial smoothing with a Gaussian kernel of 6 mm full width half maximum. Physiological artifacts and residual subject movement effects were removed through a combination of linear regression of potential confounding effects in the BOLD signal (noise components from cerebral white matter and cerebrospinal areas, estimated subject-motion parameters, identified outlier scans and task effects) and temporal band-pass filtering (0.008–0.09 Hz).

For our first-level analysis, we selected predefined networks and structures implicated in neurofeedback processing, as established by the previous literature (Emmert et al., 2016; Paret et al., 2019; Sitaram et al., 2017). These included the fronto-parietal network (lateral prefrontal cortex and posterior parietal cortex), dorsal attention (frontal eye field and intraparietal sulcus), salience network (anterior cingulate cortex, anterior insula, rostral prefrontal cortex, and supramarginal gyrus), default mode network (medial prefrontal cortex, lateral parietal and precuneus cortex), and subcortical structures (thalamus, putamen, globus pallidus, nucleus accumbens, and caudate), all defined by CONN (by default a combination of the Harvard-Oxford atlas and the AAL atlas). Then, we included weighted seed-based analysis and weighted ROI-to-ROI matrices to assess functional connectivity and its relation to the success metric as described below.

#### Seed-based connectivity.

We implemented a weighted seed-based connectivity analysis (wSBC), computed using a weighted least squares linear mode, with temporal weights corresponding to each task condition. The seed was defined as the subject-specific target ROI (DLPFC), normalized to MNI space and masked for gray matter.

The second-level analysis was based on a GLM framework (Worsley et al., 1996), taking the effect of success as the independent measure (between-subject measure) and each task condition (‘2-back’ and ‘baseline’) as the dependent measure (within-subject contrast), using a stringent initial cluster-forming height threshold set at *p* = 0.001 and an FDR-corrected *p* < 0.05 cluster-level threshold to select those clusters that are significant. Ultimately, these clusters correspond to the regions connected to the target region and to its modulation success.

#### Within-network connectivity.

Weighted ROI-to-ROI connectivity (wRRC) matrices were computed using a weighted least squares linear model, where the temporal weights correspond to each condition (‘2-back’ and ‘baseline’) and were defined as box time series convolved with a canonical hemodynamic response (Nieto-Castanon, 2020). These ROI-to-ROI metrics were extracted for the predefined networks above-mentioned (fronto-parietal network, dorsal attention, salience network, default mode network, and subcortical structures). Then, we analyze an intrinsic connectivity metric of the four networks (for a mathematical definition see the Supporting Information 1), and how it relates to the NF trend (our success metric) for each condition using a MATLAB custom script.

#### Classification scheme for success prediction based on functional connectivity.

To evaluate the predictive value of the functional connectivity data obtained from the previous correlation analyses, we developed a classification scheme based on the success of each participant. In short, we determined the target of the classification scheme as the trend of the three neurofeedback runs. If positive, the target value was set to 1, and if negative, the trend was set to zero. Nine participants were labeled as successful/learners (1) and eight participants as nonsuccessful/nonlearners (0).

The feature set was based on functional connectivity measures during the 2-back condition between ROIs integrating the DMN (intrinsic connectivity). Considering the target vector (success) and the preselected functional connectivity features, we implemented a logistic regression classifier to evaluate the predictive value of these measures. To this end, we used the scikit-learn package. To ensure robust and reliable results, we employed a leave-one-out cross-validation (LOOCV) technique, that is, fitting all participants except one and predicting out-of-sample outcomes, iteratively for each participant. The out-of-sample predictions resulting from the cross-validation were used to estimate measures of model performance. Furthermore, to validate the statistical significance of our findings, we employed permutation tests (shuffle the labels of our target vector, *n* = 1,000). By comparing the actual performance with the permutations’ distribution, we determine the significance level of our results.

### Structural Connectivity Data Analysis

First, we extracted the first b0 volume and run the brain extraction tool (BET) to create a binary brain mask image. To correct for eddy current distortion and subject motion and to detect outliers, we used eddy tool from FSL v6.0.5.2 (Smith et al., 2004). Then, the preprocessed DTI data were coregistered with T1 anatomical images in BrainSuite Software (https://brainsuite.org), using the brain diffusion pipeline (BDP), which includes INVERSION, a nonrigid registration method that uses inverted contrast relationship between T1-and T2-weighted brain, also correcting for susceptibility induced distortions (Bhushan et al., 2015). After coregistration of DWI and T1-weighted images, tensors and orientation distribution functions were estimated in each subject anatomical space using Funk-Radon and Cosine Transform (FRACT), which provides higher angular resolution than standard methods.

Anatomical data were preprocessed using the BrainSuite Anatomical Pipeline, which includes skull stripping and extraction of cortical surface, followed by surface and volume registration and labeling of subcortical and cortical structures in the subject space. We used the USCBrain atlas (Joshi et al., 2022), a high-resolution single-subject atlas based both on anatomical and functional (resting-state) information. Since our primary goal was not comparing functional and structural connectivity, we implemented an atlas at the subject space in our DTI ROI statistical analysis, which provides higher anatomical precision.

Tracts were generated using a deterministic method. ROI selection followed the same principle of functional connectivity analysis, focusing on areas that play a role in neurofeedback processing, including: middle frontal gyrus, superior parietal cortex, supramarginal gyrus, anterior and posterior cingulate gyrus, anterior insula, superior and inferior precuneus, thalamus, putamen, globus pallidus, nucleus accumbens and caudate (Emmert et al., 2016; Paret et al., 2019; Sitaram et al., 2017). Correspondence between anatomic landmarks and functional areas was verified by an expert neuroradiologist (author DJP). Here, we assumed that DLPFC would anatomically correspond to the region labeled as the middle frontal gyrus. Finally, we extracted the connectivity matrices from each subject, comprising the number of tracts passing through each pair of ROIs and normalized to its maximum element.

#### Correlation between structural connectivity and success.

Replicating the functional connectivity analysis, we performed a linear regression taking each network intrinsic connectivity (here defined as the average connectivity between all pairs of ROIs considered for each network) as a potential predictor of NF trend (our success metric). Also, to explore which specific structural connections might contribute to NF success, we modeled the relationship between all preselected paired ROIs and NF success. As a preprocessing step, we detected and removed outliers of the connectivity measure based on the interquartile range method. The data points (subjects) identified as outliers were not considered in the linear regression model between connectivity and success.

#### Classification scheme for success prediction based on structural connectivity.

We use the same classification scheme to evaluate the predictive value of the structural connectivity data. In this case the feature set was based on the subset of ROIs pairs emerging in the previous correlation analysis as significant. We extracted the number of fibers in each tract, as previously described, for each participant. Similarly, we used a LOOCV technique. Statistical significance was based on 1,000 permutation tests.

### Analysis of Structural and Functional Data Dependence

We implemented a linear regression model to test if the structural and functional connections (previously shown to be relevant for neurofeedback success prediction) influenced each other. Furthermore, we combined the features derived from functional and structural connectivity analysis in a single classifier using ridge regression, implemented in scikit-learn with regularization parameter alpha = 1.0. Again, LOOCV and permutation tests were performed for the statistical analysis of the obtained accuracy.

## RESULTS

All participants were able to modulate DLPFC activity, increasing the BOLD signal while executing the working memory imagery task and decreasing during baseline, from the initial training run, that is, even in no-feedback runs. Considering our success metric, nine participants showed improvement along the neurofeedback runs (positive slope). No relation was found between the BOLD activity level in the localizer and NF trend [*R*^2^ = 0.1, *F*(1, 15) = 1.673, *p* = 0.215].

### Functional Connectivity

#### Seed-based connectivity.

**Figure 1.**
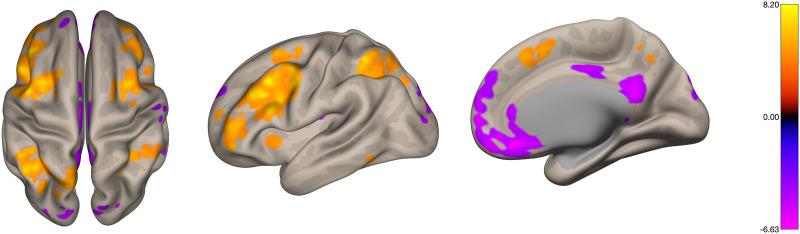
Seed-based connectivity map from all subjects, considering as seed the subject-specific DLPFC, during the 2-back condition of the localizer (*q* = 0.05, FDR corrected) showing positive clusters in the fronto-parietal network and negative clusters in the default mode network. The color bar represents the *t* value of the one-sample *t* test on the individual Fisher-transformed correlation coefficients for condition 2-back. Colored regions represent coefficients above the statistical threshold.

In Figure 1 we present a wSBC map across all subjects, with a subject-specific target region (DLPFC) seed, during ‘2-back’ condition (one-sample *t* test), showing positive clusters in the executive/fronto-parietal network including both DLPFC, other frontal related areas (namely frontal pole and precentral gyrus), superior parietal lobes and anterior/paracingulate gyrus, and negative clusters (anticorrelated) in DMN including frontal medial cortex, posterior cingulate cortex, and precuneus.

**Figure 2.**
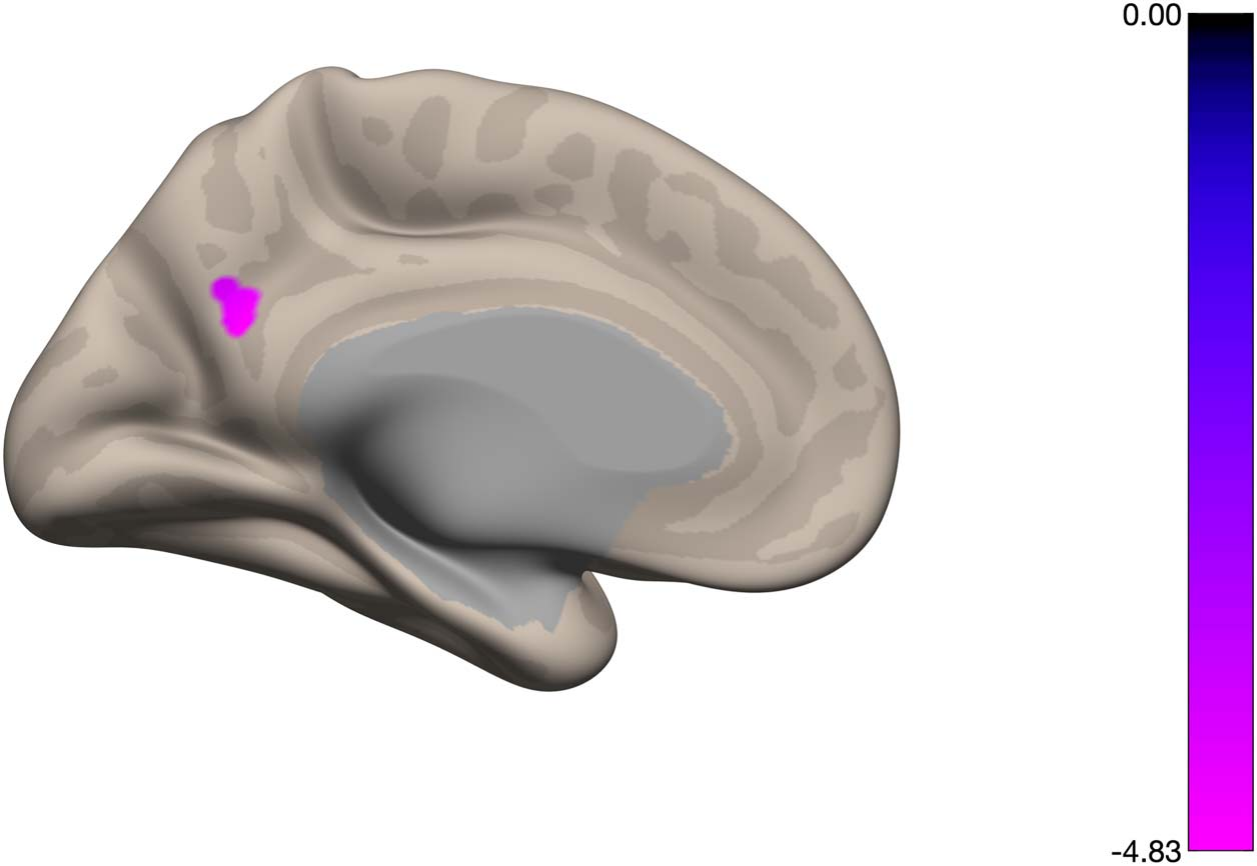
Correlation between DLPFC-based connectivity during 2-back task and success metric shows a single cluster in posterior cingulate cortex/precuneus (peak MNI coordinates: (0, −58, 24), cluster size: 31 voxels; size *p*-FWE = 0.102, size *p*-FDR = 0.04). The color bar represents regression coefficients using success as a covariate and the individual Fisher-transformed correlation coefficients for the condition 2-back. Colored regions represent coefficients above the statistical threshold.

When considering the effect of success, that is, including success of each subject as a covariate subject-level variable, we found a single cluster in inferior precuneus/PCC (peak MNI coordinates: (0, −58, 24), cluster size: 31 voxels; size *p*-FWE = 0.102, size *p*-FDR = 0.04, T = −6.58) with hypoconnectivity to DLPFC significantly correlated with the NF trend measure during the ‘2-back’ condition (Figure 2).

#### Intranetwork connectivity.

**Figure 3.**
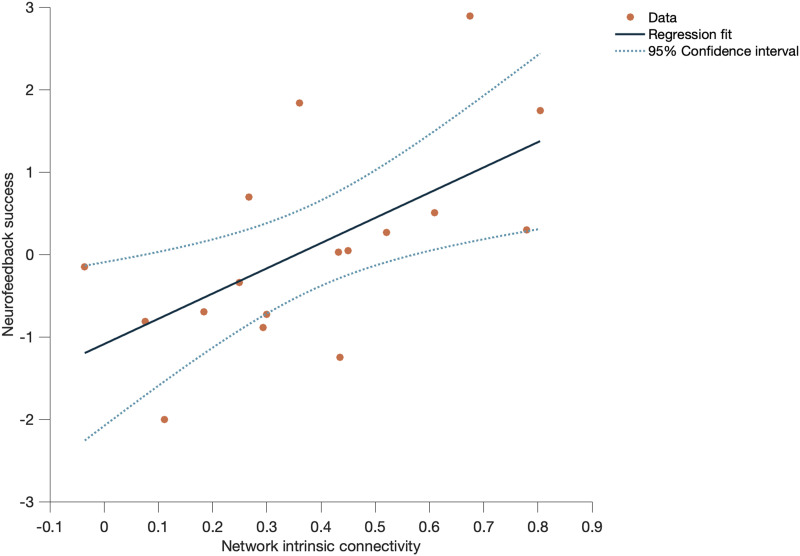
Intrinsic connectivity analysis (linear regression considering NF success as the dependent variable and ROIs mean connectivity as the predictors). Mean connectivity of the ROIs integrated within the DMN during 2-back (correlation coefficient = 0.606, *p* = 0.01).

In the investigation of main networks intrinsic connectivity implicated in neurofeedback, we found a significant positive correlation between success and the connectivity within the DMN during the 2-back condition (*R*^2^ = 0.367, *F*(1, 15) = 8.71, *p* = 0.010) (Figure 3). Correlations between intrinsic connectivity and success for the other networks were nonsignificant and are presented in Table S1 from Supporting Information 2.

#### Classification scheme for success prediction based on functional connectivity.

The features included in the model were the connectivity between ROIs within the DMN (middle prefrontal cortex, posterior cingulate cortex, and lateral parietal cortex), informed by the intranetwork correlation analysis. The LOOCV showed significant predictive value of our model, with an accuracy of 77% (*p* = 0.01, derived from 1,000 permutation tests); see features importance in Table S2 and LOOCV histogram in Figure S1 (Supporting Information 2).

### Structural Connectivity

**Figure 4.**
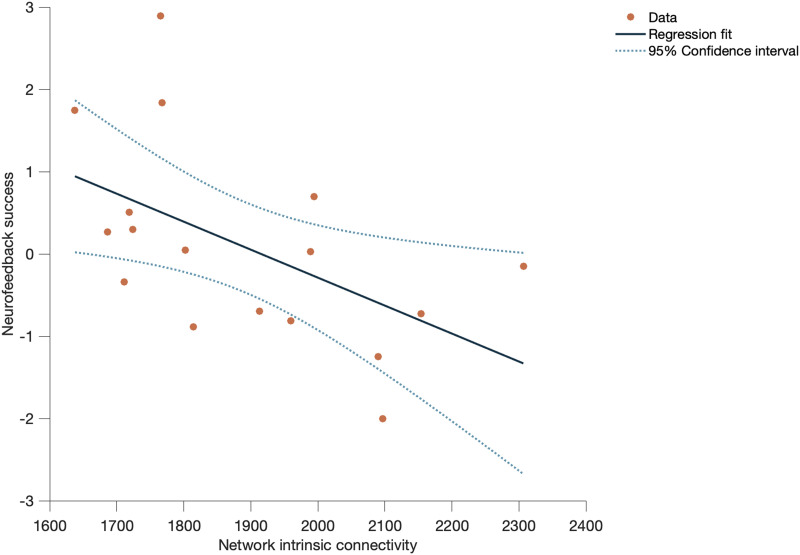
Negative correlation between NF success and average connectivity between all pairs of ROIs of salience network (correlation coefficient = −0.536, *R*^2^ = 0.2876, and *p* = 0.026).

When testing the predictive value of structural connectivity (mean number of tracts within each network) for neurofeedback success, we found a moderate negative correlation between NF success and intrinsic connectivity (average connectivity between all pairs of ROIs) of the salience network (correlation coefficient = −0.536, *R*^2^ = 0.2876, *p* = 0.026) (Figure 4).

**Figure 5.**
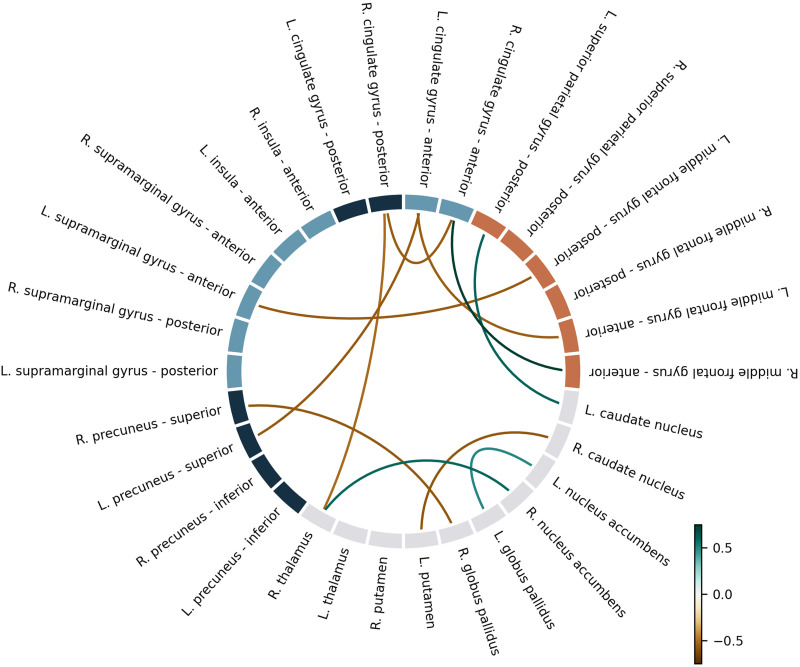
Connectivity circle representing 11 connections that show statistically significant relation between success and average number of fibers. Scale represents correlation coefficient. The right anterior middle frontal gyrus/right anterior cingulate gyrus pair was the only with a correlation coefficient superior to 0.5.

To have a better insight of the specific connections possibly contributing to the NF success predictive value, we also explored pairwise connectivity for all ROIs included in these key networks (described in detail in Table S3, Supporting Information 2). The mean number of tracts of 11 connections emerged as potential predictors of NF success (*p* < 0.05, uncorrected), which are represented in the connectivity circle (Figure 5) and also discriminated in detail in Table S4, Supporting Information 2. However, among all these correlations, the *R*^2^ value was greater than 0.5 only for the right anterior middle frontal gyrus/right anterior cingulate gyrus pair (correlation coefficient = −0.761, *R*^2^ = 0.579, *p* = 0.002).

Next, we implemented a classifier with LOOCV informed by the correlation analysis, that is, including the 11 connections that showed significant correlation between success and average number of fibers. We found a good performance of our model, predicting the NF success group (learner vs. nonlearner) with 88% accuracy (permutation test *p* value = 0.002). The five most relevant features were structural connectivity between left/right middle frontal gyrus and left/right anterior cingulate gyrus, left middle frontal gyrus and left supramarginal gyrus, left putamen and right caudate, and right thalamus and nucleus accumbens (see Table S5 and Figure S2 in Supporting Information 2).

### Structural and Functional Data Dependence

To study the relationship between the structural and functional data, we estimated the correlations (across participants) between functional and structural metrics of the significant connections in (1) functional connectivity analysis, corresponding to left lateral prefrontal cortex/middle frontal gyrus and posterior cingulate gyrus/precuneus; and (2) structural connectivity analysis, corresponding to left lateral prefrontal cortex/middle frontal gyrus and anterior cingulate cortex. No structural connections were found for the left middle frontal gyrus/precuneus pair, as expected taking into account the anatomical knowledge. For the other pair, we did not observe significant correlation between structural and functional metrics (*r* = −0.033, *n* = 15, *p* = 0.497). In what respects to the combined classifier, integrating both functional and structural features, the LOOCV showed significant predictive value with an accuracy of 88% (*p* = 0.012) (see Table S6 and Figure S2 in Supporting Information 2).

## DISCUSSION

In the present study, we investigated potential predictors of neurofeedback success (interpreted here as the trend in NF runs) based on both functional and structural connectivity metrics in a working memory paradigm targeting DLPFC. Considering network analyses on a set of networks known to be involved in neurofeedback processes, we found a positive correlation between intrinsic functional connectivity of the DMN and neurofeedback success. Moreover, a single cluster in precuneus/PCC (a well-known integrative hub of DMN) emerged from our DLPFC-based connectivity approach (i.e., seed corresponding to subject-specific NF target ROI), showing that a lower DLPFC/precuneus connectivity during the most demanding cognitive task (2-back) was predictive of better NF performance. These results are consistent with previous NF studies, targeting DLPFC and/or other EF and DMN regions/networks, that reported a predictive value for neurofeedback success of PCC/precuneus connectivity metrics from a prior resting-state fMRI (Nakano et al., 2021; Skouras & Scharnowski, 2019), but here we extend this property to the task-based localizer run, included in common neurofeedback protocols. This not only obviates the need of an additional acquisition in future studies, at least targeting EF and DMN networks/regions, but also opens the door for a replication study in a larger multicentric sample.

The DMN is anatomically defined by two main components: (1) medial prefrontal cortex, the ventral part supporting emotional processes and the dorsal linked to self-referential mental activity; and (2) PCC and medial precuneus, mainly involved in recollection of prior experiences (Buckner et al., 2008; Raichle, 2015). Moreover, it shows a well-described anticorrelation with executive control/fronto-parietal network: as the DMN activity decreases, the activity in executive control network increases, with more focused attention on the external environment, required for goal-directed behavior (Buckner et al., 2008; Fox et al., 2005; Sitaram et al., 2017). The deactivation of DMN during cognitively demanding tasks has also been proved in the context of neurofeedback, with activity decrease in PCC and precuneus (Emmert et al., 2016). In our study, DLPFC (a main component of fronto-parietal network) is the target for neurofeedback training and our results noticeably reflect this competing interaction of DMN and executive control/fronto-parietal network. These results suggest that having a less effective suppression of DMN (possibly reflected on a higher intrinsic connectivity) during a demanding working memory task, as the one performed in the localizer run before neurofeedback training, may allow more room for improvement, reflected in neurofeedback success. In other words, neurofeedback training may potentiate a more efficient regulation of the anticorrelated coupling DMN/CEN.

Considering the structural connectivity analysis, the salience network appears to play the main role in predicting NF success, particularly in its interaction with the CEN. Intrinsic connectivity of the salience network is negatively correlated with NF success. Exploring the structural connectivity for each pair of ROIs, 11 connections emerged as significant, being the main predictor the mean number of tracts between the anterior cingulate gyrus and the middle frontal gyrus (in correspondence to DLPFC). The cross-validation of the predictive model including the 11 significant connections showed an accuracy of 88%, with the connectivity between anterior cingulate gyrus and middle frontal gyrus bilaterally emerging as particularly relevant features. The salience network, mainly anchored in the anterior insula/operculum and anterior cingulate gyrus (thus, alternatively known as cingulum-operculum network), is activated in conscious perception of neurofeedback, representing a transitional network that links cognition and interoception (Dewiputri et al., 2021; Sitaram et al., 2017). The anterior cingulate cortex, particularly, is engaged in explicit response selection and conflict monitoring (Menon, 2015). It is also part of the reward system along with the nucleus accumbens-ventral striatum, which in turn has an implicit role in motivation, namely during neurofeedback processing (Pereira et al., 2023; Sitaram et al., 2017), also included in our classification model on the five most relevant features. Most important, as previously mentioned, the salience network mediates the dynamic functional switching between DMN and central executive network (Goulden et al., 2014; Sridharan et al., 2008). A previous report proved that even structural connectivity mirrors this observed dynamic switch, showing that integrity of white matter tracts in salience network is fundamental to DMN function (Bonnelle et al., 2012).

Our functional and structural predictors were not correlated. This was expected particularly for the connection between DLPFC and precuneus, taking into account the anatomical knowledge on fiber tracts, and also considering that the evaluation of correlations between the two connectivity metrics are limited by divergent ROI definition in structural and functional analysis. Nevertheless, this independence conceptually reinforces the need for a mediator for the dynamic functional switching between CEN and DMN.

Overall, functional and structural connectivity analyses complementarily and independently reflect different perspectives of the same predictive theoretical framework, in which success is partially determined by the interaction between three key networks: DMN, salience network, and central executive network. When combined in a single classification model, these functional and structural connectome features showed a significant predictive value with an 88% accuracy. Functional connectivity during a cognitive demanding task shows higher intrinsic connectivity of the DMN, but with less connectivity with the target ROI from the anticorrelated central executive network, in relation to success. This is a task-based observation, in individuals naïve to this NF paradigm, reflecting a basal working memory competence that might be improved with NF. This improvement, apparently functionally dependent on the coupling DMN/CEN networks, might be settled on the structural intrinsic characteristics of the salience network (a critical switcher).

### Limitations

The statistical power of the aforementioned correlation analysis is diminished by the modest sample size, being close to the global median of rt-fMRI neurofeedback studies (Fede et al., 2020) and justified by the exploratory character of this study. This aspect possibly impacts the results of our classification scheme, since small samples may lead to overinflation of the LOOCV accuracy, even though this is a particularly useful technique when dealing with small datasets (Varoquaux, 2018). Permutation testing improved statistical control on prediction accuracy, providing an additional estimate of the dependency between class labels and observations (Stelzer et al., 2013).

The classifications schemes were informed by the previous correlation analyses performed using the whole dataset, incurring the risk of feature leakage. On the one hand, this was partially due to the exploratory characteristics of our research, although we had an a priori hypothesis of the main networks involved. On the other hand, the modest sample size did not allow data splitting. Ultimately, we combined both structural and functional classifiers using ridge regression, which incorporates a regularization term that can help to mitigate feature collinearity and overfitting. This classifier showed to be promising, with an accuracy of 88%, although we still need to be conservative on the generalization of our model to new data.

Moreover, at this point, the interpretation of our results does not yet provide generalization also due to the specificity of the paradigm and success measure considered. However, it is critical to notice that our results in functional connectivity are in line with the two previous papers accessing resting-state functional connectomics as predictors of NF success, in which role for PCC/precuneus connectivity was proven in larger samples (Nakano et al., 2021; Skouras & Scharnowski, 2019).

Finally, our results highlight putative functional and structural independent predictors that are specific for our target (DLPFC) region and localizer task (*n*-back) and that take into account a definition of success circumscribed to the target activity during NF tasks, but not the learning effect between transfer and train runs. Therefore, we cannot specifically assess how much the measured ability to improve DLPFC activity during NF runs is influenced by motivation and the proficiency in performing a dual task (that is, the imagery task and feedback monitoring). However, those are actually the substrates for associative learning, the key for neurofeedback success, so we assume in our model their possible contribution to the functional connectivity findings.

## CONCLUSION

Dynamic switching between DMN, salience network, and central executive network seems to be the key for neurofeedback success, in DLPFC NF training, indicated by functional connectivity on a localizer run and structural connectivity data. Our study, as the first analyzing both functional and structural metrics to predict success in NF, opens the pathway to additional multimodal approaches in the field, demanding further investigation to test its reproducibility in an independent dataset, with other target regions/neurofeedback paradigms and at a larger scale.

## SUPPORTING INFORMATION

Supporting information for this article is available at https://doi.org/10.1162/netn_a_00338.

## AUTHOR CONTRIBUTIONS

Daniela Jardim Pereira: Conceptualization; Formal analysis; Investigation; Visualization; Writing – original draft; Writing – review & editing. João Pereira: Formal analysis; Methodology; Software. Alexandre Sayal: Formal analysis; Methodology; Software; Validation. Sofia Morais: Conceptualization; Investigation; Writing – review & editing. António Macedo: Investigation; Supervision; Writing – review & editing. Bruno Direito: Conceptualization; Formal analysis; Methodology; Software; Supervision; Visualization; Writing – review & editing. Miguel Castelo-Branco: Conceptualization; Data curation; Funding acquisition; Investigation; Project administration; Resources; Supervision; Validation; Writing – review & editing.

## FUNDING INFORMATION

Miguel Castelo-Branco, Fundação para a Ciência e a Tecnologia (https://dx.doi.org/10.13039/501100001871), Award ID: FCT/UIDB/4950. Miguel Castelo-Branco, Fundação para a Ciência e a Tecnologia (https://dx.doi.org/10.13039/501100001871), Award ID: FCT/UIDP/4950/2020. Daniela Jardim Pereira, Fundação para a Ciência e a Tecnologia (https://dx.doi.org/10.13039/501100001871), Award ID: SFRH/SINTD/93678/2013. Miguel Castelo-Branco, Fundação para a Ciência e a Tecnologia (https://dx.doi.org/10.13039/501100001871), Award ID: FCT/DSAIPA/DS/0041/2020. Miguel Castelo-Branco, FP7 Health (https://dx.doi.org/10.13039/100011272), Award ID: BRAINTRAIN.

## TECHNICAL TERMS

Structural connectivity: Physical pathways connecting brain regions.
Functional connectivity: Synchronized activity of different brain regions at rest or during tasks.
rt-fMRI neurofeedback: Training of brain activity self-regulation using functional neuroimaging in real time.
Seed-based connectivity: Analyzes brain connections from a chosen specific region.
Leave-one-out cross-validation: A model evaluation method performed by excluding one sample each time.
Permutation tests: Random reordering for statistical analysis validation.
