# Supplementary material for: Functional and structural connectivity success predictors of real-time fMRI neurofeedback targeting DLPFC: Contributions from central executive, salience, and default mode networks
Source: Netw Neurosci. 2024 Apr 1;8(1):81–95. doi: 10.1162/netn_a_00338 (PMC10861170; doi:10.1162/netn_a_00338)
Supplement: Supplementary file 1 [file netn-8-1-81-s001.pdf]

Supplementary Material 1

Network intrinsic connectivity Definition

The weighted adjacency matrix of a graph G is a two dimensional N x N matrix, say $A = (a_{i,j})$, where $a_{i,j}$ represents the Pearson's correlation coefficient between nodes i and j. We define intrinsic connectivity as the network cost, c, the average of the cost of each individual node, c_i, here computed as the average of the connections of each node i to all others $i \neq j$.

$$c_i = \frac{\sum_j A_{ij}}{N-1}$$

$$c = \frac{\sum_i c_i}{N}$$

Supplementary Material 2

Table S1. Correlations between intrinsic connectivity and success

Network	Condition	Ro	R^2	F(1.15)	p-value
Default Mode	baseline	-0,02	0	0,01	0,94
	2-back	0,61	0,37	8,71	**0,01**
Salience	baseline	0,4	0,16	2,87	0,11
	2-back	0,41	0,17	3,02	0,1
Dorsal Attention	baseline	0,05	0	0,04	0,84
	2-back	-0,18	0,03	0,51	0,49
Fronto-Parietal	baseline	-0,31	0,09	1,55	0,23
	2-back	-0,29	0,08	1,33	0,27
Subcortical	baseline	-0,03	0	0,02	0,91
	2-back	-0,38	0,14	2,5	0,14

Table S2. Features Importance for Logistic Regression for Functional Connectivity

Features Subset	Feature Importance
DMN.MPFC- DMN.LP (R)	1.121413
DMN.MPFC- DMN.PCC	0.821901
DMN.LP (L) - DMN.LP (R)	0.644832
DMN.LP (L) - DMN.PCC	0.549083
DMN.MPFC - DMN.LP (L)	0.511118
DMN.LP (R) - DMN.PCC	0.169799

Abbreviations: DMN – default mode network; L – left; LP – lateral parietal; MPFC – medial prefrontal cortex; PCC – posterior cingulate cortex; R - right

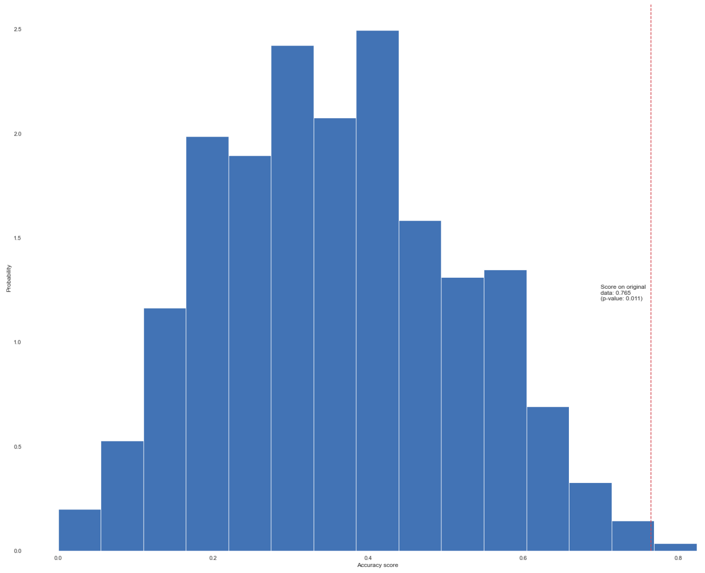

Figure S1 – Histogram of leave-one-out cross validation accuracy scores for functional connectivity classifier (permutation test p value=0.01).

Table S3. ROIs selected from USCBrain Atlas

Network	ROI			Label
Default Mode Network	anterior angular gyrus		R	236
			L	237
	posterior cingulate gyrus		R	190
			L	191
	precuneus	superior	R	254
			L	255
		inferior	R	256
			L	257
	anterior superior frontal gyrus		R	106
			L	107
Salience network	anterior cingulate gyrus		R	186
			L	187
	anterior insula		R	502
			L	503
	supramarginal gyrus	anterior	R	230
			L	231
		posterior	R	232
			L	233
Executive Network	middle frontal gyrus	anterior	R	112
			L	113
		posterior	R	114
			L	115
	superior parietal gyrus	anterior	R	244
			L	245
		posterior	R	246
			L	247
	posterior superior frontal gyrus		R	108
			L	109
Subcortical Structures	thalamus		R	640
			L	641
	putamen		R	630
			L	631
	globus pallidus		R	616
			L	617
	nucleus accumbens		R	650
			L	651
	caudate		R	620
			L	621

Table S4. Pairwise structural connectivity

Paired ROI	r	p	r^2
R ant MFG - R ant cingulate	0,761	0,002	0,579
L ant MFG - L ant cingulate	-0,561	0,024	0,315
L MFG post - L ant supramarginal	-0,573	0,026	0,328
L superior parietal - L caudate nucleus	0,611	0,027	0,373
R ant cingulate - R post cingulate	-0,565	0,018	0,319
L ant cingulate - L precuneus	-0,569	0,027	0,324
R cingulate post - R thalamus	-0,519	0,039	0,27
R precuneus sup - R GP	-0,58	0,024	0,336
R thalamus - R Nc Accumbens	0,607	0,021	0,369
R caudate - L putamen	-0,584	0,018	0,341
L GP - L Nc accumbens	0,5	0,049	0,25

Abbreviations: ant- anterior; GP – globus pallidus; L- left; MFG- middle frontal gyrus; Nc – nucleus; post- posterior; R - right

Table S5. Features Importance for Logistic Regression for Structural Connectivity

Features Subset	Feature Importance
Anterior MFG (L) - ACC (L)	1.405832
Posterior MFG (L) - Anterior supramarginal gyrus (L)	0.843440
Caudate (R) - L Putamen (L)	0.640220
Anterior MFG (R) - R ACC (R)	0.435072
Thalamus (R) - Nucleus Accumbens (R)	0.365338
PCC (R) -Thalamus (R)	0.289365
Precuneus superior (R) – Globus pallidus (R)	0.224146
Globus pallidus (L) - Nucleus Accumbens (L)	0.155006
ACC (R) – PCC (R)	0.134622
ACC (L)- Precuneus (L)	0.067272

Abbreviations: ACC – anterior cingulate cortex; L – left; PCC – posterior cingulate cortex; R – right

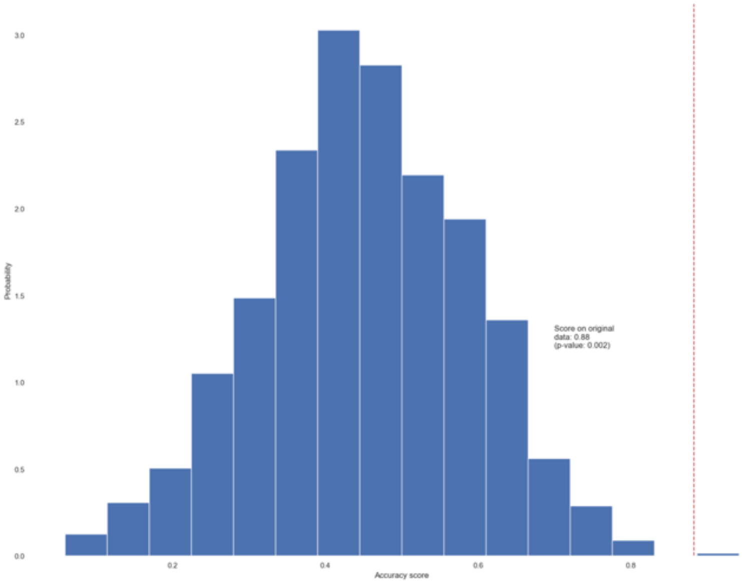

Figure S2 – Histogram of leave-one-out cross validation accuracy scores for structural connectivity classifier (permutation test p value=0.002).

Table S6. Top 5 features for Ridge Classifier combining SC and FC

Features Subset	Feature Importance
[SC] Anterior MFG (L) - ACC (L)	0.459591
[SC] Posterior MFG (L) - Anterior supramarginal gyrus (L)	0.307319
[FC] DMN.LP (L) – DMN.LP (R)	0.249892
[SC] Globus pallidus (L) - Nucleus Accumbens (L)	0.188721
[SC] Caudate (R) - L Putamen (L)	0.169402

Abbreviations: ACC – anterior cingulate cortex; DMN – default mode network; FC – functional connectivty; L – left; LP – lateral parietal; MFG – middle frontal gyrus; R – right; SC- structural connectivity

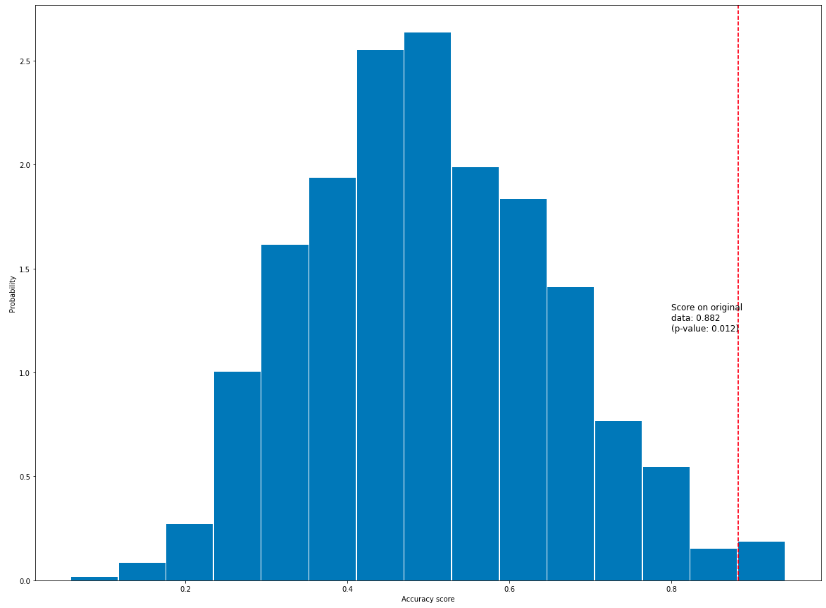

Figure S3 – Histogram of leave-one-out cross validation accuracy scores for combined functional and structural classifier (permutation test p value=0.002).
